# Whey protein lowers systolic blood pressure and Ca-caseinate reduces serum TAG after a high-fat meal in mildly hypertensive adults

**DOI:** 10.1038/s41598-018-23333-2

**Published:** 2018-03-22

**Authors:** Ágnes A. Fekete, Carlotta Giromini, Yianna Chatzidiakou, D. Ian Givens, Julie A. Lovegrove

**Affiliations:** 1 Hugh Sinclair Unit of Human Nutrition, Department of Food and Nutritional Sciences and Institute for Cardiovascular and Metabolic Research (ICMR), School of Chemistry, Food and Pharmacy, University of Reading, Reading, RG6 6AP United Kingdom; 2 Institute for Food, Nutrition and Health, University of Reading, Reading, RG6 6AP United Kingdom; 3 Department of Health, Animal Science and Food Safety, Università degli Studi di Milano, Via Trentacoste, 2, 20134 Milan, Italy

## Abstract

Epidemiological studies show an inverse association between dairy consumption and blood pressure (BP) but there are few data on the postprandial effects of milk proteins. This study examined their effects, compared to maltodextrin, on postprandial BP and other CVD risk markers in volunteers with mild and pre-hypertension over an 8 h period. In this double-blinded, randomised, cross-over, controlled study 27 adults ingested a high-fat, isoenergetic breakfast and lunch with 28 g whey protein, 28 g Ca-caseinate or 27 g maltodextrin. Whey protein reduced systolic BP compared with Ca-caseinate (−15.2 ± 13.6 mmHg) and maltodextrin (−23.4 ± 10.5 mmHg) up to 5 h post-ingestion. There was an improvement in arterial stiffness after whey protein compared with maltodextrin (incremental Area Under the Curve- iAUC_0–8h_: +14.4 ± 6.2%). Despite similar glucose levels after both whey protein and Ca-caseinate, whey protein induced a higher insulin response than Ca-caseinate (iAUC_0–8h_: +219.5 ± 54.6 pmol/L). Ca-caseinate induced less suppression of non-esterified fatty acids than whey protein (iAUC_0–5h_: −58.9 ± 135.5 μmol/L) and maltodextrin (iAUC_0–5h_: −106.9 ± 89.4 μmol/L) and induced a smaller postprandial triacylglycerol response than whey protein (iAUC_0–8h_: −1.68 ± 0.6 mmol/L). Milk proteins co-ingestion with high-fat meals may have the potential to maintain or improve CVD risk factors.

## Introduction

High blood pressure (BP) (≥140/90 mmHg) has been considered one of the major killers worldwide^1^ and is an important modifiable risk factor for CVD, among other classic risk factors such as high blood cholesterol, obesity, and smoking^2^. Arterial stiffness and endothelial dysfunction provide important information on the progression of atherosclerosis and are recognized as novel, independent risk markers of CVD^3^. Diet is a key modulator of CVD risk and epidemiological studies have identified a beneficial association between milk and dairy, particularly fermented dairy (cheese and yoghurt) consumption and CVD risk factors, specifically BP^4,5^. The particular components of milk and dairy that are associated with these reported beneficial effects are not fully elucidated, but dairy proteins and bioactive peptides have been proposed as important mediators^6,7^. In order to investigate the effects and underlying mechanisms of milk protein consumption and the vascular system, we previously performed a chronic randomised controlled dietary intervention study to investigate the long-term effects of intact milk proteins on BP and other CVD risk markers compared with carbohydrate^8^. We found that whey protein and Ca-caseinate supplementation resulted in improvements in several cardiometabolic risk factors, such as 24-h BP, arterial reactivity, blood lipids and some markers of vascular function, in fasted subjects over an 8-wk intervention period compared with a carbohydrate supplement^8^. However, it is of note that in Western countries, people spend considerable time (approximately 18 h/day) in a postprandial state, as a result of the frequent meal and snack intake. The postprandial period is considered to be an important contributing factor to chronic diseases such as atherosclerosis or diabetes^9^. Emerging evidence suggests that elevated postprandial triacylglycerol (TAG) concentrations  play an important role in the pathogenesis of CVD and is now considered as an independent risk factor for CVD^10^. Due to frequent meal consumption throughout the day, plasma TAG often does not return to fasted concentrations until early morning. Therefore, it is prudent to investigate potential dietary strategies to mitigate the metabolic disturbances that occur after food ingestion, such as hyperglycaemia and hypertriacylglycerolaemia. Whey protein has been shown to lower postprandial TAG in a limited number of RCTs^11,12^, while its effects on acute glucose metabolism have included increased plasma insulin levels and reduced peak plasma glucose compared with casein^11–17^.

Furthermore, there is a marked diurnal variation in BP among those with hypertension, with a surge in the morning, which if exaggerated, is a risk factor for stroke, independent of 24-h BP^18^. Similarly, post-waking blunting of flow-mediated dilation (FMD), a gold standard measure of macrovascular function, is an independent predictor of adverse cardiovascular events^19^. Therefore it is of clinical importance to determine whether dairy proteins can acutely reduce BP and improve vascular function, and thus exert beneficial effects during this period of increased risk. However, to the best of our knowledge, the only RCT which investigated the acute effects of dairy proteins on the cardiovascular system failed to find any acute changes in BP or augmentation index (AI) after whey protein or Na-caseinate ingestion in a 6 h postprandial study^20^.

The aim of this study was to test the hypothesis that the ingestion of milk proteins: whey protein and Ca-caseinate, with a high-fat isoenergetic breakfast and lunch will result in a reduced postprandial BP and improvements in vascular function and other CVD risk markers in adults with pre- and mild hypertension compared with carbohydrate (maltodextrin) over an 8-h postprandial period.

## Subjects and Methods

### Participants

A total of 30, healthy, non-smoking men and women with mildly elevated BP of 120/80-159/99 mmHg, aged 30–77 y, not taking antihypertensive or cholesterol-lowering medications were recruited for this trial from local communities through database, local newspapers and electronic advertisements. This study was conducted at the Hugh Sinclair Unit of Human Nutrition, University of Reading, between February 2014 and February 2015. Participants were selected if they met the inclusion criteria of BP: 120/80-159/99 mmHg, 30–77 y old, BMI 20–40 kg/m^2^, fasted glucose <7 mmol/L (not diagnosed with diabetes), cholesterol <8 mmol/L, TAG <4 mmol/L, normal liver and kidney function, and haemoglobin >110 g/dl and >140 g/dl (for women and men, respectively). Subjects provided written informed consent prior to enrolment onto the study and eligibility or exclusion was assessed by the study researcher based on a phone interview questionnaire, followed by a screening visit. Participants were excluded based on the following criteria: milk allergy or lactose intolerance, diabetes, coeliac disease, renal, gastrointestinal, respiratory, endocrine, liver disease or cancer, surgery in the previous six months, secondary hypertension, excess alcohol consumption (drinking >280 ml ethanol/wk and >210 ml ethanol/wk, for men and women, respectively), smokers, vegan, taking nutritional supplement (including fish oil, protein shakes, vitamins), anaemia, pregnancy, lactation or planning pregnancy. Blood donation three months before or during the study was not permitted. The sample size was determined from previous results from our research group^21^ and sufficient to detect a 13 mmHg difference in our primary outcome (BP) between groups with a SD of 14, at 90% power, and 5% significance level. A total of 24 participants were required to complete the study, but to account for 20% drop-out a total of 30 participants were recruited. The current study was also powered based on the study of Ballard *et al*.^22^ for the secondary outcome (FMD). To detect a 4.3% FMD intergroup difference with a SD of 2.3 at 90% power and 5% significance level, a total of six participants were required.

### Study design

The trial was conducted according to the ethical standards laid down in the 1964 Declaration of Helsinki and the Research Ethics Committee at the University of Reading (Research Ethics Committee Project No. 12/40) gave approval for the study to be conducted. All experiments were performed in accordance with the relevant guidelines. The study was registered with the clinicaltrials.org (NCT02090842, registered on 17th June 2013). This trial was a randomised, double-blinded, controlled, three-way cross-over, acute dietary intervention study with a 2-wk run-in period prior to the beginning of the trial. The study design is presented in Supplementary Figure 1. The volunteers were randomised by an independent researcher using an Excel-based randomisation program^23^, who was also responsible for treatment allocation. Equal numbers of volunteers were allocated to six treatment sequences (ABC, ACB, BAC, BCA, CAB and CBA). A double-blinded protocol was maintained throughout the study, during sample analysis and statistical analysis, the codes for the study drinks were kept under lock at Volac Int. Ltd. (Cambridge) and University of Reading.

The primary outcome was postprandial, 8 h BP, secondary outcomes were vascular reactivity measured by FMD, changes in plasma lipids, markers of insulin resistance, arterial stiffness measured by pulse wave analysis (PWA) and digital volume pulse (DVP).

### Study drinks and test meals

The test meals consisted of a breakfast (at time 0) and a lunch (at time 330 min). The breakfast included 75 g croissants (All Butter Mini Croissants, ASDA), 15 g butter (ASDA), four pieces of shortbread fingers (Paterson’s); the lunch consisted of 125 g white-bread (Kingsmill Soft White Bread), 25 g butter (ASDA), 40 g Philadelphia soft cheese spread (Kraft Foods Limited). The test meals were isoenergetic and each of them also consisted of either 28 g whey protein (Volac Int Ltd, Cambridge) or Ca-caseinate (Garret Ingredients, Thornbury); or 27 g maltodextrin (carbohydrate; MyProtein, Northwich), which were mixed with 250 ml low-nitrate water (Buxton Mineral water, Nestlé Waters UK) and 0.5 g sugar-free vanilla flavour (Myprotein, Northwich). For nutrient composition of the study drinks, see our previous publication^8^, and for the acute test meal nutrients composition, see Supplementary Table 1, which was based on the DISRUPT study^24^ with higher protein content in this trial.

### Study visits

The detailed protocol for the screening visit has been published elsewhere^8^. Two weeks prior to the first intervention study day, all participants attended a familiarisation visit (Visit 0) at the Hugh Sinclair Unit of Human Nutrition in order to familiarise volunteers with the environment and reduce the ‘white-coat effect’. This was a one-on-one session with the study researcher who thoroughly explained the procedure of the intervention visits and the requirements for the day before the study days. All vascular techniques and their implementation were clearly explained and demonstrated to the participants. Volunteers were asked to refrain from alcohol consumption and strenuous exercises 24-h before visits, and at the commencement of each study visit they had to orally confirm compliance to this protocol. Participants consumed a low-fat and low-protein meal the evening before visits that was provided to them and drank only low-nitrate water (Buxton Mineral water, Nestlé Waters UK). On the first study visit, participants drank ad libitum Buxton water. The time and amount of water consumption was recorded and the same amount was given at the same time on the following study visits in order to standardise water intake throughout the study. During acute study visits, upon arrival at the Unit, body weight and composition were measured as specified previously^8^. Participants then rested for 30 min in a supine position in a quiet, temperature-controlled room (20 °C ± 1) prior to vascular testing. All measurements were conducted by the same researcher (except automated DVP, which was taken by multiple trained researchers). These measurements were repeated on three occasions during the day. After the baseline vascular measurements and before breakfast, a cannula was inserted into the antecubital vein in the left arm and a baseline blood sample was taken. Blood samples were then collected at 30-min intervals for 2 h and every 60 min for a further 3 h. After lunch (time 330 min) blood was taken every half an hour for 1.5 h and then 60 min for an hour. Participants returned for the second and third arm of the study after a 3–4 wk washout period each. Premenopausal women attended all visits at the same phase of their menstrual cycle.

### BP and assessments of vascular function

BP was measured on the left arm using a non-invasive automatic BP monitor (TM-2430; A&D Ltd) according to the manufacturer’s instructions. The cuff-size was selected based on upper arm circumference measurements. Participants rested for at least 1 h before the BP monitors were fitted by one researcher (after baseline vascular measurements taken) and were removed after 8 h. All baseline BP measurements were taken in triplicate, after which the monitor was programmed to take the BP readings automatically at 15 min intervals for a total period of 8 h. The vascular function tests were taken after the volunteers rested for 20 mins and in the following order: DVP, PWA, FMD at time 0, time 180 min, 300 min and 420 min. For further details on these assessments, see our previous publication^8^.

### Biochemical analyses

Serum separator blood tubes were centrifuged at 1800 × g for 15 min at 20 °C (VACUETTE, Greiner Bio-One, UK); lithium heparin and ethylenediaminetetraacetic acid (EDTA) blood collection tubes (VACUETTE, Greiner Bio-One, UK) were centrifuged at 1800 × g for 10 min at 4 °C. Samples were kept at −20 °C for further analyses. TAG, glucose and non esterified fatty acids (NEFA) were quantified in serum by using an autoanalyser (ILAB600, Werfen (UK) Ltd, Warrington, UK; reagents and analyser: Instrumentation Laboratory Ltd.; NEFA reagent: Alpha Laboratories). An ELISA kit was used to determine serum insulin (Dako Ltd.). Serum angiotensin-converting enzyme (ACE) activity was determined by a fluorometric assay as described elsewhere^25^.

### Statistical analyses

Data were analysed using IBM SPSS Statistics version 21 and SAS version 9.4 (SAS Institute). Data checks for normality were performed using histograms and Q-Q plots using SPSS. The data from participants’ who withdrew from the study were excluded in the analysis as a per protocol analysis was used. The primary analysis of the data was by repeated measures ANOVA to identify significant time × treatment interactions with Bonferroni correction to control for multiple comparisons using SPSS. As secondary data analysis, area under the curve (AUC) was calculated by the trapezium rule, which was subtracted from the fasting value to calculate incremental AUC (iAUC). AUC were analysed by linear mixed-model ANOVA using the difference from fasting values as dependent variable and baseline values of the variable of interest, BMI, age, sex and intervention treatment as prognostic variables by SAS. P ≤ 0.05 was considered significant, two-tailed p-value was used. Data presented in the text, tables, and figures represent the arithmetic means ± SEMs.

## Results

### Participants

**Figure 1:**
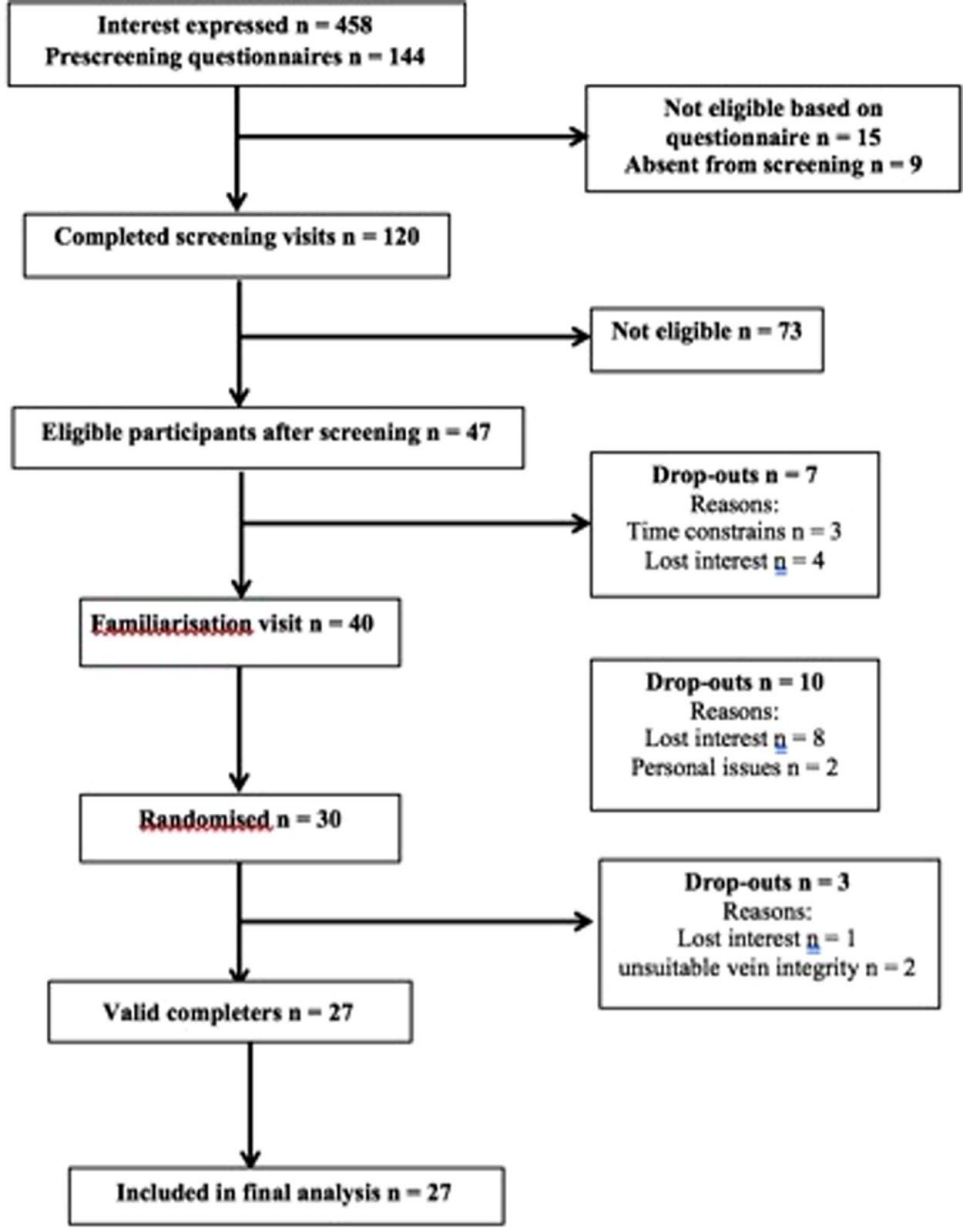
Participants inclusion flow-chart of the acute Whey2Go study.

A total of 30 people were randomised to the study, of which three withdrew (n = 1 lost interest, n = 2 unsuitable vein integrity) (see Fig. 1. for flow-chart). The baseline characteristics of completing participants can be seen in Table 1. The study drinks were well tolerated, however two volunteers discontinued the Ca-caseinate study-arm right before lunch due to feeling full and not being able to consume the second meal. Therefore data were collected from n = 27 volunteers until lunch (5 h), and n = 25 up to 8 h. The cannula failed on two participants in one of the study arms, therefore blood sample data were excluded from those participants, thus collecting data from n = 23 for 8 h and n = 26 until lunch (5 h) for blood analyses, apart from for ACE activity (which was significantly influenced by haemolysis), where n = 22.

**Table 1:** Baseline characteristics of participants^1^.

N, male/female	27 (24/3)
Age, y	50.1 ± 2.3
BMI, kg/m^2^	27.3 ± 0.7
SBP, mmHg	133.2 ± 2.4
DBP, mmHg	81.5 ± 2.1
TC, mmol/L	5.09 ± 0.2
TAG, mmol/L	1.38 ± 0.1
Glucose, mmol/L	5.44 ± 0.1

^1^BMI, body mass index; DBP, diastolic blood pressure; SBP, systolic blood pressure; TC, total cholesterol; TAG, triacylglycerol. Mean ± SEM.

### Postprandial BP

**Figure 2:**
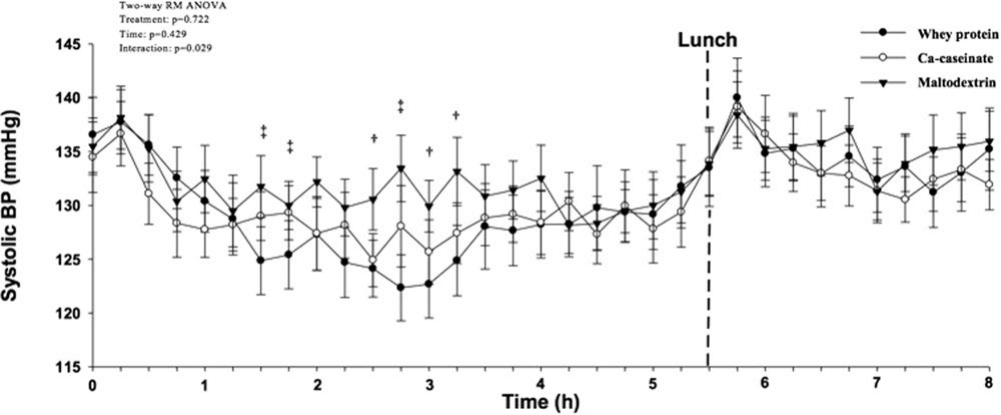
8-h postprandial BP in men and women with pre- and mild hypertension after consuming whey protein, Ca-caseinate and maltodextrin. Values are means ± SEM, n = 25. ^‡^Whey protein vs. Ca-caseinate and maltodextrin p < 0.005; ^†^Whey protein vs. maltodextrin p < 0.005; BP, blood pressure; ACE, angiotensin-converting enzyme; iAUC, incremental area under the curve; RM, repeated-measure.

There were no treatment or time × treatment effects on BP, pulse pressure and heart rate for the 8 h period. However there was a significant overall time × treatment effect (p = 0.029) on systolic BP (SBP) until 5 h (Fig. 2). Similarly, a significantly lower iAUC for SBP was observed for the consumption of whey protein compared with maltodextrin (p = 0.039) 5 h post-ingestion (Table 2).

**Table 2:** BP, vascular function measures and serum biochemistry of participants with pre- and mild hypertension at baseline, iAUC_0–5h_, and iAUC_0–8h_ after whey protein, Ca-caseinate and maltodextrin consumption1.

	Baseline			iAUC_0–5h_			p-value^2^	iAUC_0–8h_			p-value^2^
	Whey protein	Ca-aseinate	Maltodextrin rate	Whey protein	Ca-caseinate	Maltodextrin		Whey protein	Ca-caseinate	Maltodextrin	
SBP, mmHg	136.6 ± 3.5	134.5 ± 3.5	135.5 ± 2.6	−26.9 ± 9.6^a^	−11.6 ± 12.7^a,**b**^	−3.5 ± 5.9^b^	0.039	−49.8 ± 15.0	−32.4 ± 19.3	−19.3 ± 9.1	0.101
DBP, mmHg	83.3 ± 2.5	81.5 ± 2.0	84.0 ± 2.3	−24.5 ± 4.6	−15.3 ± 5.6	−17.1 ± 5.6	0.117	−43.4 ± 7.0	−29.1 ± 7.9	−36.6 ± 9.0	0.182
PP, mmHg	51.8 ± 2.2	52.9 ± 2.3	48.7 ± 1.7	2.7 ± 8.6	2.9 ± 8.6	26.7 ± 8.1	0.696	7.35 ± 12.7	−3.1 ± 14.2	32.7 ± 11.6	0.342
Heart rate, b/min	59.5 ± 2.0	59.0 ± 2.1	59.6 ± 1.9	32.7 ± 4.1	32.9 ± 4.3	−31.1 ± 2.7	0.623	43.7 ± 6.5	44.9 ± 5.8	40.8 ± 4.4	0.844
AI@HR75, %	12.2 ± 2.2	10.8 ± 2.8	11.7 ± 2.7	−18.2 ± 2.7^a^	−13.8 ± 3.0^a,**b**^	−9.1 ± 2.9^b^	0.028	−30.9 ± 4.4^a^	−22.0 ± 4.9^a,**b**^	−16.7 ± 4.5^b^	0.043
SI, %	8.15 ± 0.4	8.35 ± 0.5	8.15 ± 0.4	−0.75 ± 1.3	−2.71 ± 0.9	0.61 ± 0.7	0.065	−0.67 ± 2.1	−3.82 ± 1.5	0.24 ± 1.1	0.125
Glucose, mmol/L	5.10 ± 0.1	5.08 ± 0.1	5.03 ± 0.1	2.05 ± 0.3^a^	1.87 ± 0.4^a^	4.03 ± 0.7^b^	<0.001	4.40 ± 0.6^a^	4.08 ± 0.7^a^	9.74 ± 1.4^b^	<0.001
Insulin, pmol/L	49.04 ± 5.3	45.26 ± 4.3	43.90 ± 4.6	767.6 ± 70.6^a^	649.4 ± 63.2^b^	887.5 ± 83.6^c^	<0.001	1346.6 ± 112.8^a^	1127.1 ± 111.4^b^	1482.7 ± 150.4^a^	<0.001
NEFA, μmol/L	475 ± 45	496 ± 43	505 ± 37	−1015 ± 162^a^	−956 ± 147^b^	−1063 ± 135^a^	0.027	−1337 ± 324	−1256 ± 284	−1488 ± 278	0.112
TAG, mmol/L	1.55 ± 0.2	1.49 ± 0.2	1.47 ± 0.2	3.57 ± 0.4^a^	2.78 ± 0.3^b^	2.94 ± 0.4^a^	0.026	8.10 ± 0.8^a^	6.43 ± 0.7^b^	5.96 ± 0.8^b^	<0.001
ACE activity, U/L	4.82 ± 0.2	4.61 ± 0.2	4.48 ± 0.2	−0.02 ± 0.4	0.41 ± 0.7	1.44 ± 0.7	0.338	−0.26 ± 0.6	0.71 ± 1.1	2.23 ± 1.3	0.398

^1^Values are means ± SEM; n = 25 on vascular function measures for 8-h post-ingestion, and n = 27 on vascular function measures for 5-h post-ingestion, n = 23 on blood biochemistry for 8-h post-ingestion, and n = 26 on blood chemistry for 5-h post-ingestion. Baselines were significantly different (p ≤ 0.05) from one another, apart from glucose for 5-h post-ingestion and FMD both for 5-h and 8-h post-ingestion. Different superscript letters within a row refers to treatment groups different from one another, p ≤ 0.05. All data were logtransformed apart from FMD, AI@HR75 and SI. AI@HR75, augmentation index when corrected for a heart rate of 75 mm Hg DBP, diastolic blood pressure; NEFA, non-esterified fatty acid; PP, pulse pressure; SBP, systolic blood pressure; SI, stiffness index; TAG, triacylglycerol; Δ change from baseline.

^2^Overall between-group treatment effects for each Δ obtained by using linear mixed-model ANOVA with baseline values for the variable of interest, and prognostic values such as age, gender, BMI. Tukey-Kramer’s correction was used as post hoc anaysis to adjust for multiple testing. The values with difference superscript letters are significantly different from each other.

### Vascular function

**Figure 3:**
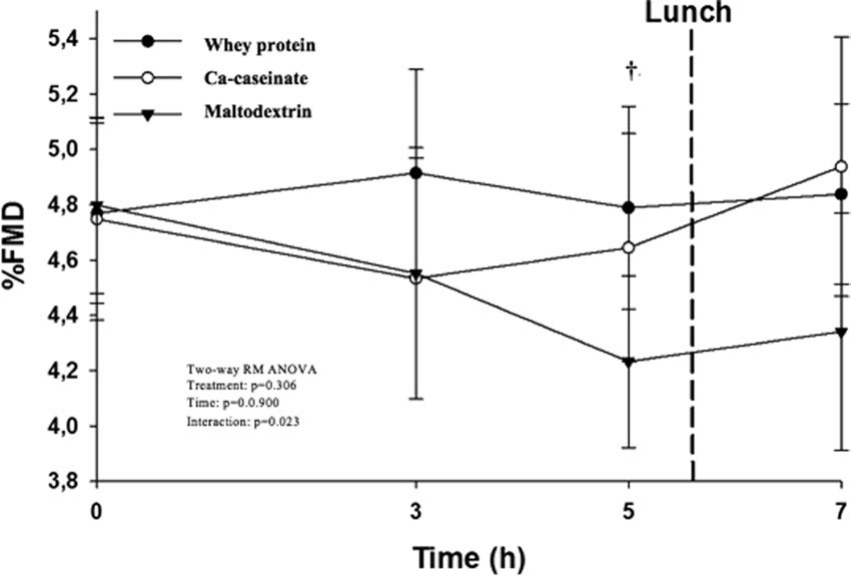
8-h postprandial FMD response in men and women with pre- and mild hypertension after consuming whey protein, Ca-caseinate and maltodextrin. ^†^p = 0.017 between whey and maltodextrin at 5-h post-ingestion according to post hoc test (one-way repeated-measures ANOVA). Values are means ± SEM, n = 25. FMD, flow-mediated dilation.

There was a time × treatment effect on FMD 8 h post-ingestion (p = 0.014). Whey protein significantly increased %FMD at 5 h after breakfast compared with maltodextrin (p = 0.017) (Fig. 3). The iAUC for AI measured by PWA decreased after whey protein ingestion compared with maltodextrin both after 5 h (p = 0.028) and 8 h (p = 0.043). There were no treatment or time × treatment effects on peripheral and central systolic, diastolic or mean pressure; however there was a tendency for significant time × treatment effect: Ca-caseinate lowered central SBP compared with maltodextrin 8 h post-ingestion (p = 0.052). No effect was seen on the stiffness index of DVP (Table 2).

### Serum glucose, insulin and lipids

**Figure 4:**
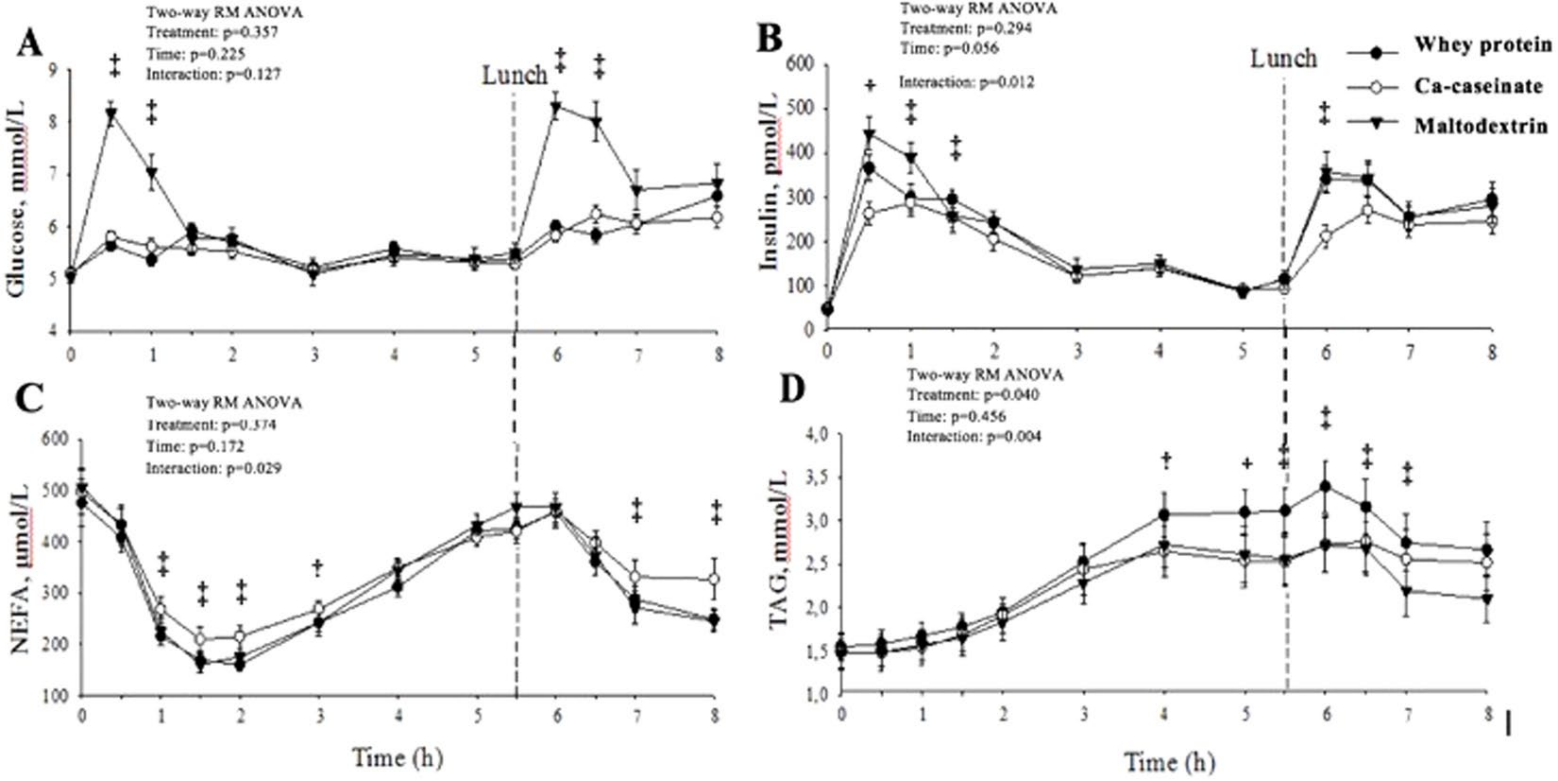
8-h postprandial glucose (**A**), insulin (**B**), NEFA (**C**) and TAG (**D**) response in men and women with pre- and mild hypertension after consuming whey protein, Ca-caseinate and maltodextrin. Values are means ± SEM, n = 23. h, hour; NEFA, non-esterified fatty acid; TAG, triacylglycerol. (**A**) ^‡^Maltodextrin vs. whey protein and Ca-caseinate p < 0.001. (**B**) ^‡^At 30 min whey protein vs. Ca-caseinate p < 0.001, whey protein vs. maltodextrin p = 0.003, maltodextrin vs. Ca-caseinate p < 0.001; at 60 min whey protein vs. maltodextrin p = 0.015, Ca-caseinate vs. maltodextrin p < 0.001; at 90 min whey protein vs. Ca-caseinate p = 0.014, whey protein vs. maltodextrin p = 0.049; at 360 min whey protein vs Ca-caseinate p < 0.001, Ca-caseinate vs. maltodextrin p = 0.001. (**C**) ^‡^Ca- caseinate vs. whey protein and maltodextrin p < 0.05. (**D**) ^‡^Whey protein vs. Ca-caseinate and maltodextrin p < 0.05.

The 8 h postprandial serum glucose, insulin and lipid responses after whey protein, Ca-caseinate and maltodextrin co-ingestion with a high-fat meal are presented in Fig. 4. The iAUC for glucose was lower after whey protein and Ca-caseinate ingestion compared with maltodextrin (5 and 8 h post-ingestion, p < 0.001) (see Table 2). The iAUC for insulin increased following maltodextrin and whey protein consumption compared with Ca-caseinate at 5 h and 8 h post-ingestion (p < 0.001). The iAUC for NEFA showed treatment effect (p = 0.027) until 5 h: postprandial NEFA levels decreased between breakfast and lunch, the least suppression was seen after Ca-caseinate ingestion compared with whey protein and maltodextrin. No treatment or time × treatment effects were seen 8 h post-ingestion. There were significant treatment (p = 0.040) and time × treatment effects (p = 0.004) and differences in iAUC (p = < 0.001) for TAG where whey protein increased TAG concentration compared with Ca-caseinate and maltodextrin both 5 h and 8 h post-ingestion.

### ACE activity

There was no treatment effect in iAUC or time × treatment interactions either 8 h or 5 h post-ingestion (Table 2).

## Discussion

People in Western countries spend the majority of their time in a postprandial state, which is considered to be an important contributing factor to chronic diseases such as atherosclerosis and diabetes^9^. This novel study investigated whether an isoenergetic, high-fat breakfast and lunch consumed with either whey protein, Ca-caseinate compared with a carbohydrate source (maltodextrin), improved postprandial BP, vascular function, glucose, and lipid metabolism. We found that whey protein significantly decreased SBP 5 h post-ingestion compared with Ca-caseinate (−15.2 ± 13.6 mmHg) and carbohydrate (−23.4 ± 10.5 mmHg). Only one previous RCT evaluated the acute effects of milk proteins on BP, and their results were in contrast with ours. They found no effects of dairy proteins on BP^20^. The authors stated that the neutral results may have been due to the high fat content of their study meal (45% of total energy), yet our breakfast contained similar fat content (46% of total energy). However, even though they administered 45 g of whey protein or Na-caseinate at breakfast as opposed to 28 g of milk proteins in our study, it is important to point out that Pal and Ellis^20^ used normotensive participants. It is therefore not surprising that no decrease in BP was observed in the previous study, as baseline BP has been shown to be an important moderator of hypotensive effects of milk proteins^6,26^.

One of the potential mechanisms of action that has been proposed for the hypotensive effects of milk proteins is ACE inhibition. Previously we reported that whey protein permeate expressed significantly higher ACE inhibitory activity *in vitro* compared with Ca-caseinate and maltodextrin^8^. Although we failed to identify treatment effects in serum ACE activity in this study, the direction of treatment effects whey>casein>maltodextrin is supportive of the postprandial BP change. It is of note that high inter-individual variation was observed and greater study power may have been required to detect significant changes in serum ACE activity after milk protein ingestion, and should not be ruled out as a potential mechanism of action. Further research should aim to elucidate this issue. Whey protein attenuated the reduced vasodilation measured by FMD at 3 h post-ingestion, which was observed after Ca-caseinate and maltodextrin ingestion. At 5 h post-ingestion, FMD was significantly higher after whey protein ingestion compared with maltodextrin; whereas Ca-caseinate induced a gradual improvement in endothelial function between 3 and 5 h, and showed similar response to whey protein at 5 h post-ingestion. This may be due to the slower-release and absorption of Ca-caseinate, as opposed to whey protein, which is a so-called ‘fast-release’ protein, being rapidly absorbed into the circulation^27^. Soop *et al*. suggested that the time-course of milk protein absorption should be considered, as casein may offer some advantages including prolonged appearance of circulating amino acids, while whey protein is rapidly eliminated from the circulation^28^. It is speculative that Ca-caseinate may also have similar beneficial effects on endothelial function acutely, as seen in our chronic trial^8^, however somewhat delayed. A longer postprandial period is required to confirm this. Ballard *et al*. investigated the acute effects of a whey protein hydrolysate (NOP-47) in older individuals^22^. They found transient improvements in FMD (4.3 ± 0.5%) compared with placebo 120 min post-ingestion^22^, while we found only 0.60% improvements 300 min after breakfast. However, it is of note that the high-fat breakfast in the current study may have exerted significant deleterious effect on the endothelium and delayed the effect of whey protein due to slower gastric emptying.

Interestingly, despite the neutral effects of long-term whey protein consumption on arterial stiffness, whose measure is AI, in our chronic study^8^, we found a significant decrease of AI postprandially after whey protein ingestion compared with carbohydrate. In contrast, Pal and Ellis failed to show any effects of milk proteins on AI in their acute study^20^, however they reported a significant decrease of AI after 12-wk intervention that included 2 × 27 g/d whey protein consumption compared with carbohydrate^29^. It is of note that, although AI is a measure of arterial stiffness (an independent predictor of cardiovascular risk), in a postprandial study it provides an estimation of central haemodynamics assessed by the shape of the pulse^30^. It has been demonstrated that AI can change postprandially, and this may be due to altered arterial smooth muscle relaxation in the splanchnic bed in response to food intake, leading to neural or hormone-mediated smooth muscle relaxation in the general circulation^31^. Interestingly, previous research showed that insulin production after food intake in a postprandial setting is the main determinant of arterial stiffness^32,33^. This is underpinned by our finding that whey protein induced increased insulin response, compared to Ca-caseinate, and similar to the magnitude observed after maltodextrin ingestion, especially after the second test meal, yet the maltodextrin did not reduce AI. Therefore, there may be other underlying mechanisms by which whey protein affects AI, which warrants further research. Furthermore, we observed similar serum glucose responses after whey protein and Ca-caseinate consumption, compared to expected elevated responses after maltodextrin, which is in contrast with other studies, where whey protein induced a smaller glucose response compared with casein^12,34^. However, whey protein ingestion resulted in similar serum insulin concentrations to maltodextrin (iAUC 0–8 h whey: 1346.6 ± 112.8, iAUC 0–8 h maltodextrin: 1482.7 ± 150 pmol/L) both being significantly higher than after Ca-caseinate ingestion. Whey protein is considered as an insulin-secretagogue, which may be due to its effects on pancreatic β-cells^35^ and/or incretin hormones^36^. Likewise, whey protein has a high branch-chained amino acid content and because of its fast absorption rates, triggers rapid and high insulin secretion. Insulin is intimately involved with lipolysis^37^, which may explain why whey protein and maltodextrin resulted in a greater NEFA suppression 5 h after breakfast ingestion compared with Ca-caseinate. This may be a consequence of the slower rate of Ca-caseinate absorption and thus a less rapid suppression of lipolysis through reduced insulin release. This finding is in contrast with a study of Holmer-Jensen *et al*. in which a smaller NEFA suppression was observed after a meal containing whey protein^11^, and with Bendtsen and co-workers’ study where NEFA concentration was higher after whey protein ingestion compared with intact and hydrolysed casein^38^. Furthermore, surprisingly we found significantly higher circulating TAG concentrations after whey protein ingestion compared with Ca-caseinate and maltodextrin. Elevated postprandial TAG is considered a CVD risk factor and appears to be more discriminatory than fasting TAG^10^. Yet, high postprandial TAG after whey protein ingestion in our trial did not seem to impact detrimentally on BP or vascular function. In contrast, previous RCTs found lowering of postprandial TAG after whey protein consumption compared with casein or other dietary proteins such as cod^12,39^. Nevertheless, postprandial TAG response does not provide a detailed insight into postprandial lipoprotein metabolism and other biomarkers such as apolipoproteins and particle number (evaluated by apo B-48 response) and size which may have provided a more complete evaluation of the postprandial effects of the interventions. However, it is puzzling that long-term whey protein consumption was found to decrease fasting TAG in our chronic study^8^, yet we report increased postprandial TAG concentrations  in this postprandial trial. It is of note that the beneficial effect of Ca-caseinate on TAG may be due to its high Ca content (more than 2.7-fold higher than whey protein). Evidence suggests that Ca can interact with fatty acids, particularly saturated fatty acids within the gut and speculatively forming insoluble soaps and/or formation of insoluble aggregation with phosphorus and biles acids and fatty acids, which are resistant to digestion and are subsequently excreted in the faeces^40^. These have been proposed as potential mechanisms by which dairy products, particularly cheese which contains high Ca levels, can reduce circulatory lipid concentrations^41^.

A potential limitation of this study is the lack of the measurements of faecal fat and Ca excretion, which would have helped to elucidate whether this potential mechanism could have affected postprandial lipid metabolism. Maltodextrin was used as a source of carbohydrate in this study as it had a lack of flavour, easy dissolvability and acceptable to the participants. However postprandial responses may have varied if different types of carbohydrate were used (such as starch or glucose). Moreover, if a test meal with a different nutritional composition was consumed, the acute resonses may have differed from that reported in this study. The dairy proteins used in this study were unhydrolysed and in a form similar to that found in dairy foods, it would therefore be predicted that the equivalent quantity of dairy protein consumed as a food would have elicited similar vascular effect as the isolated dairy proteins, however this would need confirmation in further studies. Furthermore, the current trial was not powered to detect changes in serum ACE activity so the lack of significant effect of this analyte may have been a consequence of small sampe size.

### Perspectives

We observed a significant fall in SBP after 28 g whey protein co-ingestion with an isoenergetic, high-fat breakfast compared with Ca-caseinate and maltodextrin in individuals with pre- and mild hypertension. Concomitantly, whey protein appeared to improve measures of postprandial arterial stiffness throughout the 8 h postprandial period compared with maltodextrin. Furthermore, although whey protein consumption did not improve endothelial-dependent vasodilation significantly compared with baseline, or the other interventions; whey protein significantly attenuated the reduction in FMD compared with Ca-caseinate and maltodextrin ingestion. Whey protein induced a similar insulin response and NEFA suppression, to maltodextrin, but a lower glucose response. Whereas Ca-caseinate ingestion resulted in significantly lower insulin response which was reflected in a reduced NEFA suppression compared with whey protein and maltodextrin, and induced lower postprandial TAG concentrations compared with whey protein. Further research should elucidate the underlying mechanism of the short- and long-term effects of whey protein on postprandial AI, insulin and TAG.

## Electronic supplementary material

Supplementary file
