# Supplementary material for: Whey protein lowers systolic blood pressure and Ca-caseinate reduces serum TAG after a high-fat meal in mildly hypertensive adults
Source: Sci Rep. 2018 Mar 22;8:5026. doi: 10.1038/s41598-018-23333-2 (PMC5864936; doi:10.1038/s41598-018-23333-2)
Supplement: Supplementary file 1 — Supplementary file [file 41598_2018_23333_MOESM1_ESM.pdf]

**Whey protein lowers systolic blood pressure and Ca-caseinate reduces serum TAG after a high-fat meal in mildly hypertensive adults.**

Ágnes A. Fekete[1,2], Carlotta Giromini[3], Yianna Chatzidiakou[1], D. Ian Givens[2], Julie A. Lovegrove[1,2*]

[1] Hugh Sinclair Unit of Human Nutrition, Department of Food and Nutritional Sciences and Institute for Cardiovascular and Metabolic Research (ICMR), School of Chemistry, Food and Pharmacy, University of Reading, Reading RG6 6AP, United Kingdom

[2] Institute for Food, Nutrition and Health, University of Reading, Reading RG6 6AP, United Kingdom

[3] Department of Health, Animal Science and Food Safety, Università degli Studi di Milano, Via Trentacoste, 2, 20134 Milan, Italy

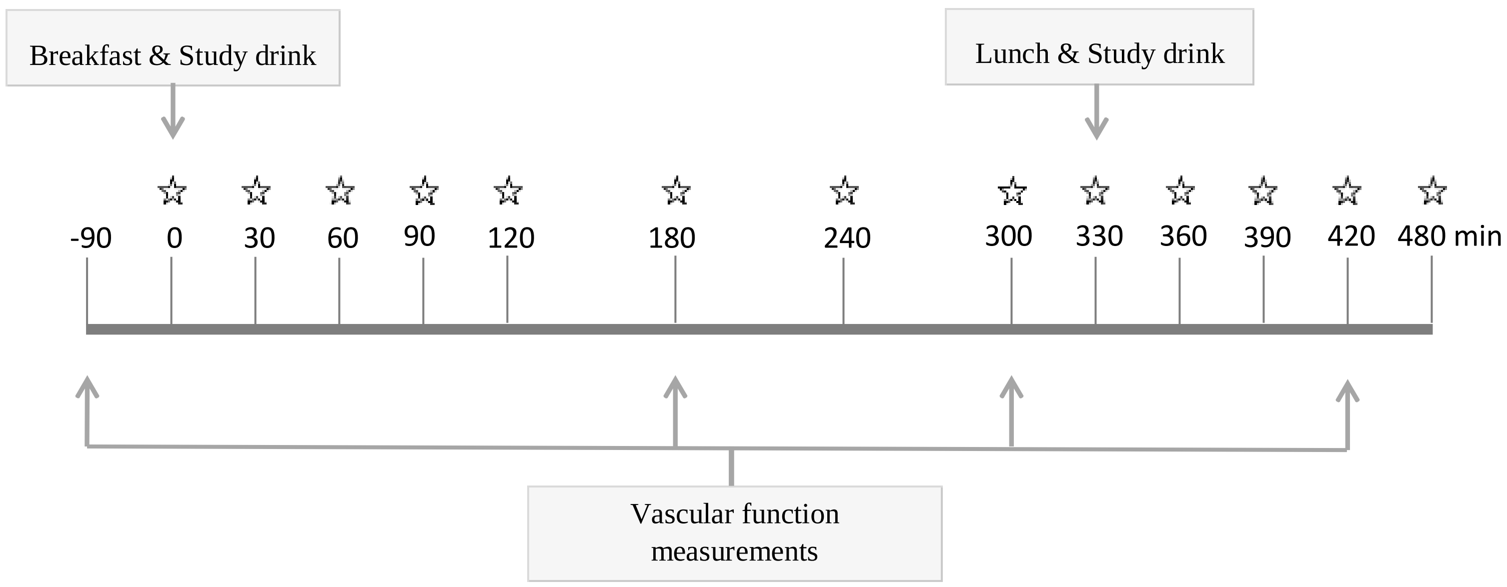


**Supplementary Figure 1.** Acute study design for the Whey2Go study. Stars denote for blood withdrawal

**Supplementary Table 1.** Nutritional composition of test meals for the acute Whey2Go study[1]

| | Whey protein | | Ca-caseinate | | Maltodextrin | |
|---|---|---|---|---|---|---|
| | Breakfast | Lunch | Breakfast | Lunch | Breakfast | Lunch |
| Energy, MJ | 4.2 | 2.6 | 4.2 | 2.6 | 4.2 | 2.6 |
| Protein, g, (%TE) | 37 (15%) | 37 (24%) | 37 (15%) | 37 (24%) | 12 (5%) | 12 (8%) |
| Carbohydrate, g, (%TE) | 95 (38%) | 46 (30%) | 95 (38%) | 46 (30%) | 120 (48%) | 71 (46%) |
| Fat, g, (%TE) | 52 (46%) | 32 (46%) | 52 (46%) | 32 (46%) | 52 (46%) | 32 (46%) |
| SFA, g, (%TE) | 25 (22%) | 18 (26%) | 25 (22%) | 18 (26%) | 25 (22%) | 18 (26%) |

[1] MJ, megajoule; SFA, saturated fatty acid; %TE, percentage of total energy.

**Supplementary Table 2.** The nutrient contribution of the study supplements in two sachets a day

| Nutritional information | Whey protein | Ca-caseinate | Control |
|---|---|---|---|
| Energy (kJ) | 893.8 | 850.6 | 879.1 |
| Protein (TN × 6.38) (g) | 49.6 | 50.2 | 0.1 |
| Total carbohydrate (g) | 2.9 | <0.01 | 51.4 |
| Fat (g) | <0.1 | 0.4 | 0.1 |
| Saturates (g) | <0.1 | 0.2 | <0.1 |
| Monounsaturates (g) | <0.1 | 0.1 | <0.1 |
| Polyunsaturates (g) | <0.1 | <0.1 | <0.1 |
| Trans fatty acids (g) | <0.1 | <0.1 | <0.1 |
| Moisture (g) | 2.,7 | 3.9 | 2.4 |
| Ash (g) | 1.7 | 2.6 | <0.01 |
| Sodium (mg) | 160 | 13 | <2.7 |
| Calcium (mg) | 235 | 650 | 0.49 |
| Magnesium (mg) | 37 | 5.4 | <0.05 |
| Potassium (mg) | 319 | 6.2 | 0.9 |
| Phosphorus (mg) | 129 | 448 | <2.7 |
| Amino acid composition (g) | | | |
| Aspartic acid | 4.75 | 2.68 | - |
| Threonine | 4.39 | 2.19 | - |
| Serine | 2.13 | 2.14 | - |
| Glutamic acid | 7.95 | 8.51 | - |
| Proline | 2.54 | 3.94 | - |
| Glycine | 0.61 | 0.60 | <0.001 |
| Alanine | 2.00 | 1.02 | <0.001 |
| Cysteine | 0.95 | 0.42 | - |
| Valine | 2.31 | 2.26 | <0.001 |
| Methionine | 0.99 | 1.12 | - |
| Iso-leucine | 2.70 | 1.81 | <0.001 |
| Leucine | 4.33 | 3.40 | <0.002 |
| Tyrosine | 1.19 | 2.02 | - |
| Phenylalanine | 1.27 | 1.96 | - |
| Histidine | 1.00 | 1.37 | <0.016 |
| Lysine | 4.17 | 3.00 | <0.001 |
| Arginine | 0.77 | 1.36 | - |

This table is previously published in Fekete et al. AJCN. 2016, doi: 10.3945/ ajcn.116.137919.
